# Body image and behavioural and emotional difficulties in German children and adolescents

**DOI:** 10.1186/s12887-023-04405-3

**Published:** 2023-11-23

**Authors:** Lea Krause, Tanja Poulain, Wieland Kiess, Mandy Vogel

**Affiliations:** 1 Department of Women and Child Health, University Hospital for Children and Adolescents, Center for Pediatric Research (CPL), Leipzig University, Liebigstrasse 20a, Leipzig, 04103 Germany; 2 LIFE Leipzig Research Center for Civilization Diseases, Leipzig University, Philipp-Rosenthal-Strasse 27, Leipzig, 04103 Germany

**Keywords:** Body image satisfaction, Body size perception, Behavioural difficulties, Emotional difficulties

## Abstract

**Background:**

Behavioural and emotional difficulties might play an important role in the development of body image disturbances, which represent serious risk factors for eating disorders or depression. The present study provides a detailed overview on body image disturbances and several behavioural and emotional difficulties (differences between gender, age, and weight status) and their inter-relations in German children and adolescents.

**Methods:**

Data on body image disturbances, assessed through a Figure Rating Scale, and on behavioural and emotional difficulties, assessed through Goodman’s Strengths and Difficulties Questionnaire (SDQ), were available for 5255 observations of 1982 German children and adolescents aged 8 to 18 years from the LIFE Child study, based in Leipzig, Germany. Associations were investigated using multiple logistic regression. Each association was checked for interaction with gender, age, and weight status.

**Results:**

Boys reported more behavioural difficulties than girls, while girls reported more emotional difficulties. Gender, age and weight status were related to behavioural and emotional difficulties as well as body image disturbances. Individuals with fewer difficulties were more satisfied with their own body. Children and adolescents who desired to be larger showed more prosocial behaviour problems, conduct and emotional problems and more signs of hyperactivity. Those, who desired to be thinner showed more problems in all SDQ-subscales. A more accurate body size perception was associated with fewer behavioural and emotional difficulties. Children and adolescents who overestimated their body size showed more prosocial behaviour and emotional problems. Underestimation one’s body size was associated with more signs of hyperactivity.

**Conclusion:**

The current findings highlight the importance of raising the awareness about the association between behavioural and emotional difficulties and body image disturbances in children and adolescents to prevent negative outcomes.

**Supplementary Information:**

The online version contains supplementary material available at 10.1186/s12887-023-04405-3.

## Background

The body image is a multidimensional concept incorporating a person’s perception, thoughts and feelings regarding his/her own body. Body image dissatisfaction describes the feelings resulting from the mismatch between the perception of one’s body (i.e., body image) and one’s ideal body. The body size perception relates to an (in)accurate perception of one’s body size [12].

The development of a negative body image can be explained by Thompson’s Tripartite Influence Model (1999) [58]. Influenced by media, peers, and family, the model suggests that people become aware of body ideals. Especially the increasing usage of social media over the last years leads to thin-ideal internalization and appearance comparison with others, which eventually leads to body image dissatisfaction [1, 22]. Also parental criticism about the children’s weight or look has a huge impact on the development of body image dissatisfaction [1, 34]. Furthermore, parents who are concerned about their weight and diet promoting dissatisfaction among their children by being a role model [34]. The comparison of one’s appearance to suggested body ideals leads to body image disturbances in children and adolescents which are serious risk factors for weight loss behaviours such as skipping meals or excessive exercising, and eating disorders [23, 52]. Over the last decade, body positivity has become more present in social media, emphasizing body acceptance and promoting different beauty standards and diverse body types [15]. However, researcher showed that body positivity was more likely to promote public health concerns like overweight and obesity and their psychological and physical health consequences [15, 44].

The body image is also influenced by various sociodemographic factors, like age or gender. Since adolescence is a transition stage marked by physical and psychological changes, it is a particularly vulnerable period for the development of body dissatisfaction [13, 50]. Therefore, it is not surprising that older children and adolescents are more dissatisfied with their body than younger children or adults [36, 53].

Influenced mainly by media, girls desire a thin body size, whereas boys strive for a more muscular body, which could lead to body dissatisfaction in both girls and boys [22]. Moreover, the peer pressure combined with constant social comparison stimulates the internalization of an unrealistic and, therefore, unachievable body ideal [57].

Previous research indicated that body image dissatisfaction is stronger in girls than boys [36, 46]. Additionally, Riahi et al. (2019) found that girls tend to overestimate and boys to underestimate their body weight [47]. However, other studies could not find a gender difference [2, 3].

Recent studies also found body dissatisfaction and body weight misperception associated with the weight status. Children and adolescents with overweight or obesity were more dissatisfied [23] and desired a thinner body than normal weight children and adolescents [26, 40].

Regarding emotional difficulties, body dissatisfaction and body weight misperception might increase the risk for depression and anxiety in children and adolescents [6, 21, 63].

However, according to another study, body weight misperception in children and adolescents with overweight or obesity seemed to be a protective factor for depressive symptoms [59].

Regarding behavioural difficulties, children and adolescents who reported hyperactivity or conduct problems also showed greater body dissatisfaction [10, 46].

Although several studies have investigated associations between body dissatisfaction and emotional or behavioural difficulties in children and adolescents, most of them have focused on only one difficulty or one disease (i.e. ADHD, depression). Furthermore, associations between the discrepancy of the self-perceived body size and the actual body weight (body size misperception) and emotional or behavioural difficulties have been understudied.

Therefore, our study aims to describe the distributions of body image (dis-)satisfaction and body size (mis-)perception in a German childhood cohort and examines their associations with behavioural and emotional difficulties in healthy children and adolescents under consideration of age, gender, and weight status. Compared to boys, we hypothesized girls to show more emotional and less behavioural difficulties, to be more dissatisfied, and to report a higher body size misperception. We also expected children and adolescents who perceived their body size correctly, to be more satisfied with their body image. Furthermore, we hypothesized that emotional and behavioural difficulties correlate positively with body dissatisfaction and body size misperception.

## Methods

### Participants

Data were collected within the LIFE Child study, a longitudinal cohort study located in Leipzig, Germany. Since 2011, this study recruits infants, children and adolescents without chronic, chromosomal or syndromal diseases from i.e., kindergartens, schools or public health centers to study determinants of healthy development as well as risk factors for civilization diseases. Participants go through assessment once every year. LIFE Child consists of three interrelated cohorts: the birth cohort, the health cohort, and the obesity cohort. The LIFE Child study has been described in detail elsewhere [41, 45].

We included all 8- to 18-year-old children and adolescents from the health cohort and the obesity cohort with completed Strengths and Difficulties Questionnaire (SDQ) and a valid Figure Rating Scale (FRS) who participated at least once between 2011 and 2021. Our sample consisted of 5255 observations from 1982 children and adolescents (1017 male (51%), 965 female (49%)). Participants themselves (over the age of 18 years) or parents provided informed written consent before participation [41, 45].

The study was designed under the supervision of the Ethics Committee of the University of Leipzig (Reg. No. 264/10-ek). It was performed in accordance with the ethical standards as laid down in the 1964 Declaration of Helsinki and its later amendments. All participants were informed about the study program and the long-term use of data.

### Measures

To assess the children’s body mass index (BMI), height and weight were measured by trained research assistants. Subsequently, BMI values were transformed into standard deviation scores (BMI-SDS) using the German age- and gender-specific norms [30]. Following the guidelines of the German Obesity Society, weight groups were defined as: underweight (BMI-SDS ≤ -1.28), normal weight (-1.28 < BMI-SDS ≤ 1.28), overweight (1.28 < BMI-SDS ≤ 1.88) and obese (BMI-SDS > 1.88) [37].

**Fig. 1:**
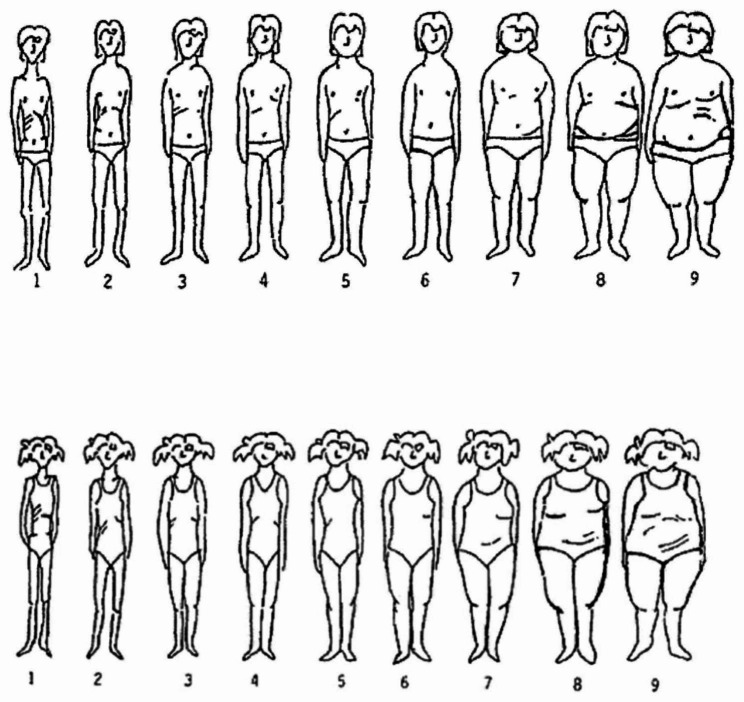
Figure Rating Scale for boys and girls

A Figure Rating Scale (FRS) based on Stunkard’s Figure Rating Scale (1983) [56] and Collins Figure Rating Scale (1991) [16] was used to assess the self-perceived body size and the desired body size of the children and adolescents (see Fig. 1). This scale was adapted to the needs of LIFE Child. It contains nine silhouettes ranging from very thin (a value of 1) to obese (a value of 9). Here, silhouette 1 corresponds to children and adolescents being underweight, silhouettes 3 to 5 to being normal weight, silhouette 6 to being overweight and silhouettes 8 and 9 to being obese. Silhouettes 2 and 7 are intermediate stages between underweight-normal weight (silhouette 2) or between overweight-obese weight (silhouette 7). Participants were asked to pick the silhouette representing (a) their current body size and (b) their desired body size.

Body (dis-)satisfaction [26, 38, 40] was calculated as the difference between the desired and the current body size score. A zero discrepancy corresponds to the participant’s satisfaction with his/her current body size; a positive/negative value corresponds to the wish to be thinner/larger. Furthermore, a difference of ± 1 is interpreted as slightly dissatisfied, a difference of ± 2 as moderately dissatisfied and a difference of > + 2 or < -2 as very dissatisfied [38].

To investigate the body size (mis-)perception, the discrepancy between the actual weight status derived from the BMI-SDS and the weight status corresponding to the perceived body size (Figure Rating Scale) was analyzed. Again, a zero discrepancy indicated that the participant perceived his/her body size right; if the discrepancy had a positive/negative value, the participant over-/underestimated his/her body size. The discrepancy was dichotomized into (a) correct perception vs. misperception, (b) perception as too thin vs. other, and (c) perception as too large vs. other.

Behavioural and emotional difficulties were assessed using the German version of Goodman’s Strengths and Difficulties Questionnaire (SDQ) [25, 65]. It is an internationally validated, standardized screening questionnaire for children and adolescents and consists of 5 scales, each containing 5 items: prosocial behaviour (Cronbach’s alpha = 0.69), hyperactivity (Cronbach’s alpha = 0.7), emotional problems (Cronbach’s alpha = 0.71), conduct problems (Cronbach’s alpha = 0.52) and peer relationship problems (Cronbach’s alpha = 0.58). The description of each subscale has been described elsewhere [24]. All items are rated on a three-point Likert scale (0 = not true, 1 = somewhat true, 2 = certainly true) resulting in scores ranging from 0 to 10 for each scale. For all scales, except for prosocial behaviour, a higher score indicates greater difficulties. On the prosocial behaviour scale, 5 is considered a borderline state and $\le$4 abnormal. On the hyperactivity and emotional problem scales, 6 is considered a borderline state and $\ge$7 abnormal. On the conduct problem scale, 4 is considered a borderline state and $\ge$5 abnormal. On the peer relationship problem scale, 4 and 5 are considered a borderline state and $\ge$6 abnormal [25]. In the present study, borderline and abnormal state are combined and interpreted as showing symptoms. Moreover, only the sum of each scale was analyzed. We applied the parent-report version for the 8-10-years-old children and the youth self-report version for the 11-18-years-old children.

### Statistical analysis

Data analysis was performed using R version 4.1.1. Descriptive statistics are given as mean and standard deviation (SD) for continuous variables and as count and percentages for categorical variables. Internal consistency for the SDQ subscales was calculated as Cronbach’s alpha.

To examine associations of each SDQ-scale and the discrepancies of the body image disturbances as dependent variables with gender, age and BMI-SDS as independent variables, multiple hierarchical regression was used. The subject was added as random effect to account for multiple observations per subject.

Associations of body satisfaction, the desire to be thinner/larger, an accurate body size perception and over-/underestimation (as dependent variables) with each SDQ-scale (as independent variable) were assessed by multiple logistic regressions. Further, to investigate the associations between the directions and degree of discrepancy, we modeled SDQ-scales non-linearly dependent on the discrepancy using Poisson regression. Every statistical model was checked for interactions between the independent variables and age or gender.

To investigate the association of body size perception as dependent variable with body satisfaction as independent variable, multiple logistic regressions were used.

Effects were reported as odds ratios (OR) and the respective 95%-confidence interval (95% CI). All analyses were adjusted for gender, age and BMI-SDS, if appropriate.

## Results

### Distribution of body image disturbances and association between behavioural and emotional difficulties and body image disturbances with sociodemographic factors

For better comparability, the distributions of body image disturbances are presented separately for girls (49.0%) and boys (51.0%), for three age groups (8-10-year-old (29.7%), 11-13-year-old (36.7%) and 14-18-year-old (33.6%)) and for the four weight groups (underweight (8.1%), normal weight (71.7%), overweight (8.1%) and obesity (12.1%)), as shown in Table 1. An additional file shows the distributions of behavioural and emotional difficulties (see Additional file 1). An overview of the associations between behavioural and emotional difficulties and body image disturbances with gender, age and BMI-SDS are listed in Table 2.

**Table 1:** Distributions of body image disturbances

	female (n = 2562)	male (n = 2693)	8–10 years (n = 1563)	11–13 years (n = 1929)	14–18 years (n = 1763)	Underweight (n = 427)	Normal weight (n = 3767)	Overweight (n = 426)	Obese (n = 635)
Body satisfaction									
satisfied	857 (33.5%)	1230 (45.7%)	771 (49.3%)	719 (37.3%)	597 (33.9%)	186 (43.6%)	1809 (48.0%)	58 (13.6%)	34 (5.35%)
slightly dissatisfied	939 (36.7%)	923 (34.3%)	487 (31.2%)	700 (36.3%)	675 (38.3%)	145 (34.0%)	1421 (37.7%)	168 (39.4%)	128 (20.2%)
moderately dissatisfied	470 (18.3%)	340 (12.6%)	181 (11.6%)	311 (16.1%)	318 (18.0%)	70 (16.4%)	395 (10.5%)	114 (26.8%)	231 (36.4%)
very dissatisfied	296 (11.6%)	200 (7.43%)	124 (7.93%)	199 (10.3%)	173 (9.81%)	26 (6.09%)	142 (3.77%)	86 (20.2%)	242 (38.1%)
Desire									
to be thinner	1496 (58.4%)	1067 (39.6%)	605 (38.7%)	1011 (52.4%)	947 (53.7%)	29 (6.79%)	1571 (41.7%)	364 (85.4%)	599 (94.3%)
to stay the same	857 (33.5%)	1230 (45.7%)	771 (49.3%)	719 (37.3%)	597 (33.9%)	186 (43.6%)	1809 (48.0%)	58 (13.6%)	34 (5.35%)
to be larger	209 (8.16%)	396 (14.7%)	187 (12.0%)	199 (10.3%)	219 (12.4%)	212 (49.6%)	387 (10.3%)	4 (0.94%)	2 (0.31%)
Body size perception									
Overestimation	254 (9.91%)	362 (13.4%)	138 (8.83%)	265 (13.7%)	213 (12.1%)	361 (84.5%)	215 (5.71%)	40 (9.39%)	0 (0.00%)
No misperception	1755 (68.5%)	1858 (69.0%)	1024 (65.5%)	1301 (67.4%)	1288 (73.1%)	66 (15.5%)	3108 (82.5%)	181 (42.5%)	258 (40.6%)
Underestimation	553 (21.6%)	473 (17.6%)	401 (25.7%)	363 (18.8%)	262 (14.9%)	0 (0.00%)	444 (11.8%)	205 (48.1%)	377 (59.4%)

**Table 2:** Association between behavioural and emotional difficulties and body image disturbances with sociodemographic factors

	Age		Gender^b^		BMI-SDS	
	OR (95% CI)	p-value	OR (95% CI)	p-value	OR (95% CI)	p-value
Prosocial behaviour^a^	0.94 (0.87–1.01)	0.08	2.94 (1.72–5.05)***	< 0.001	1.16(0.95–1.40)	0.14
Hyperactivity^a^	0.86 (0.82–0.90)***	< 0.001	1.56 (1.16–2.10)**	0.003	1.04 (0.93–1.17)	0.45
Emotional problems^a^	1.09 (0.99–1.19)	0.58	0.36 (0.18–0.71)**	0.003	1.16 (0.91–1.48)	0.22
Conduct problems^a^	0.71 (0.66–0.77)***	< 0.001	1.68 (0.96–2.93)	0.07	1.5 (1.22–1.85)***	< 0.001
Peer relationship problems^a^	1.04 (0.99–1.09)	0.06	1.18 (0.88–1.57)	0.27	1.69 (1.51–1.90)***	< 0.001
Body satisfaction	0.90 (0.87–0.93)***	< 0.001	2.11 (1.70–2.61)***	< 0.001	0.40 (0.36–0.44)***	< 0.001
desire to be thinner	1.12 (1.07–1.16)***	< 0.001	0.22 (0.17–0.29)***	< 0.001	8.14 (6.87–9.64)***	< 0.001
desire to be larger	1.04 (0.99–1.10)	0.15	2.88 (2.00-4.15)***	< 0.001	0.12 (0.09–1.55)***	< 0.001
Accurate body size perception	1.09 (1.05–1.13)***	< 0.001	1.06 (0.85–1.33)	0.58	0.86 (0.80–0.95)**	0.002
overestimation	1.10 (1.01–1.19)*	0.02	1.60 (0.84–3.04)	0.15	0.18 (0.13–0.26)***	< 0.001
underestimation	0.81 (0.77–0.85)***	< 0.001	0.67 (0.5–0.89)**	0.007	2.60 (2.27–2.97)***	< 0.001

^a^ showing symptoms; ^b^ reference: female

OR = Odds Ratio, CI = Confidence Intervall

*** = p < 0.001; ** = p < 0.01; * = p < 0.05

Boys showed significantly more prosocial behaviour problems (OR = 2.94, p < 0.001) and hyperactivity (OR = 1.56, p < 0.01) but fewer emotional problems (OR = 0.36, p < 0.01) than girls. The differences in gender for conduct problems and peer relationship problems (all p > 0.05) were not significant.

Older children and adolescents showed fewer symptoms of hyperactivity (OR = 0.86, p < 0.001) and conduct problems (OR = 0.71, p < 0.001), whereas prosocial behaviour problems, emotional problems and peer relationship problems were not significantly associated with age (all p > 0.05).

Children and adolescents with higher BMI-SDS showed significantly more symptoms of conduct problems (OR = 1.50, p < 0.001) and peer relationship problems (OR = 1.69, p < 0.001). For prosocial behaviour, hyperactivity and emotional problems, no significant association with BMI-SDS could be found (all p > 0.05).

Overall, 60% of all children and adolescents were at least slightly dissatisfied with their body image. Boys were significantly more satisfied with their body image than girls (OR = 2.11, p < 0.001). Moreover, body satisfaction was significantly higher with younger age (OR = 0.90, p < 0.001) and a decreasing BMI-SDS (OR = 0.40, p < 0.001), with normal weight (48.0%) and underweight (43.6%) children and adolescents being the most satisfied.

Compared to girls, boys desired less frequently to be thinner (39.6%, OR = 0.22, p < 0.001), but more frequently to be larger (14.7%, OR = 2.88, p < 0.001). Additionally, the desire to be thinner was significantly associated with older age (OR = 1.12, p < 0.001) and higher BMI-SDS (OR = 8.14, p < 0.001), whereas the desire to be larger was only significantly related to lower BMI-SDS (OR = 0.12, p < 0.001) but not to age (p = 0.15).

Regarding accurate body size perception, 68% of all children and adolescents perceived their body size correctly, with no significant difference between girls (68.5%) and boys (69%, p = 0.58). Accurate body size perception was significantly associated with older age (OR = 1.09, p < 0.001) and lower BMI-SDS (OR = 0.86, p < 0.01), with normal weight children and adolescents (82.5%) having the most accurate body size perception. Underweight children and adolescents (15.5%) showed the least accurate body size perception.

Overestimating one’s body size was significantly associated with older age (OR = 1.10, p = 0.02) and lower BMI-SDS (OR = 0.18, p < 0.001), whereas underestimation was significantly associated with younger age (OR = 0.81, p < 0.001) and higher BMI-SDS (OR = 2.60, p < 0.001). Compared to girls, boys were significantly less likely to underestimate their body size (OR = 0.67, p < 0.001). There was no significant difference between genders found for overestimation (p = 0.15).

### Association between body image satisfaction and body size perception

**Fig. 2:**
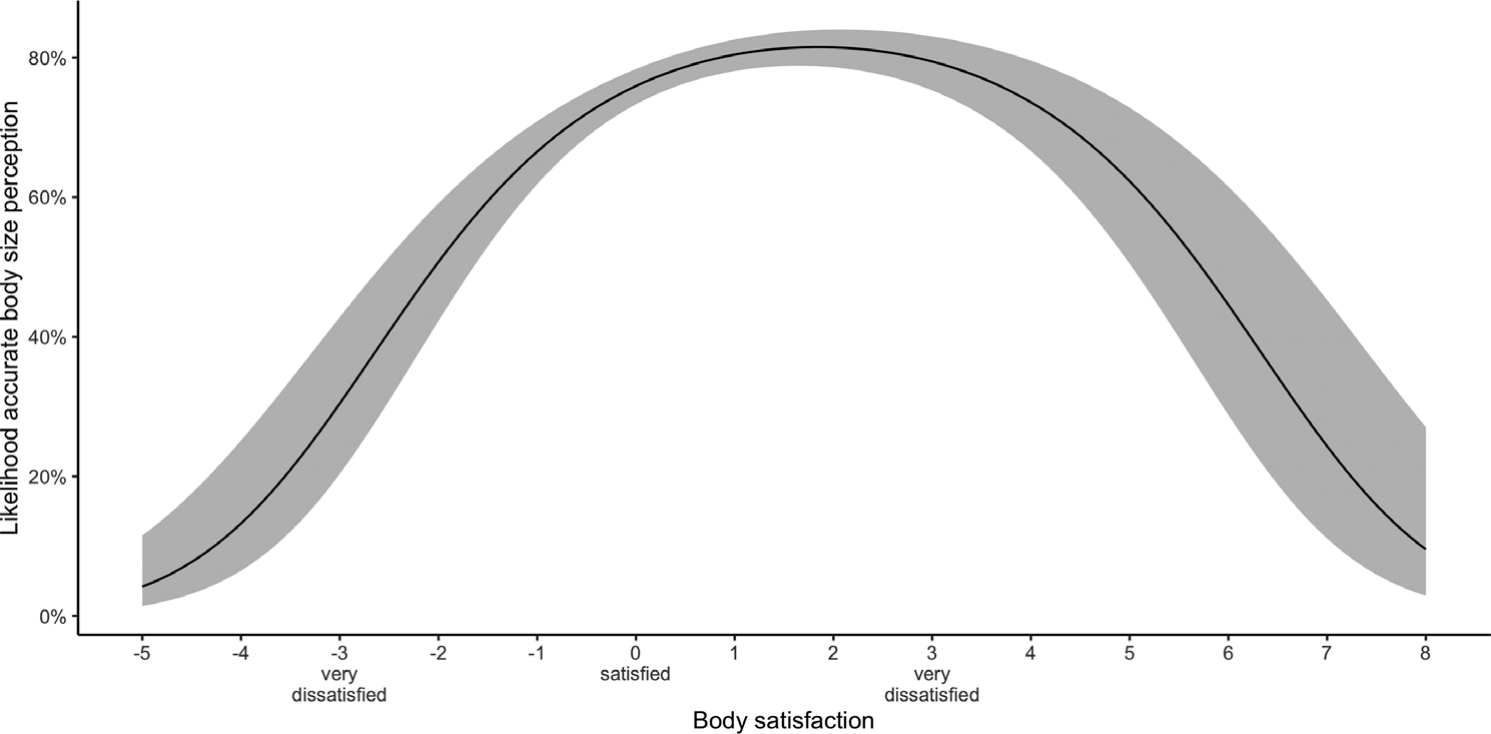
Association between body satisfaction and the likelihood for an accurate body size perception. The shaded area indicates the 95% confidence intervals of the estimated effect

As shown in Fig. 2, the likelihood of perceiving the accurate body size decreased significantly with a higher body image dissatisfaction (OR = 0.70, p < 0.001). However, children and adolescents who desired to be thinner and were moderately dissatisfied were more likely to perceive their body size correctly than children and adolescents who desired to be larger and were also moderately dissatisfied.

The model was checked for interactions between each independent variable and age or gender. However, interactions did not reach statistical significance.

### Association between behavioural and emotional difficulties and body image disturbances

As shown in Table 3, body satisfaction was positively associated with prosocial behaviour (OR = 1.15, p < 0.001) and negatively associated with hyperactivity (OR = 0.88, p < 0.001), conduct problems (OR = 0.84, p < 0.001), emotional problems (OR = 0.84, p < 0.001) and peer relationship problems (OR = 0.87, p < 0.001).

The desire to be thinner was significantly associated with more prosocial behaviour problems (OR = 0.88, p < 0.001) and more signs of hyperactivity (OR = 1.09, p < 0.001), conduct problems (OR = 1.12, p = 0.002), emotional problems (OR = 1.14, p < 0.001) and peer relationship problems (OR = 1.11, p = 0.002).

Similarly, the desire to be larger was significantly associated with more problems in prosocial behaviour (OR = 0.91, p = 0.04), and more signs of hyperactivity (OR = 1.17, p < 0.001), conduct problems (OR = 1.23, p < 0.001) and emotional problems (OR = 1.16, p < 0.001). However, no significant association was found between the desire to be larger and peer relationship problems (p = 0.08).

**Table 3:** Association between behavioural and emotional difficulties and body satisfaction

SDQ-subscale^a^	Body Satisfaction		Desire to be thinner		Desire to be larger	
	OR (95% CI)	p-value	OR (95% CI)	p-value	OR (95% CI)	p-value
Prosocial behaviour	1.15 (1.09–1.21)***	< 0.001	0.88 (0.83–0.94)***	< 0.001	0.91 (0.84–0.99)*	0.04
Hyperactivity	0.88 (0.84–0.91)***	< 0.001	1.09 (1.04–1.14)***	< 0.001	1.17 (1.10–1.26)***	< 0.001
Emotional problems	0.84 (0.83–0.88)***	< 0.001	1.14 (1.08–1.20)***	< 0.001	1.16 (1.08–1.25)***	< 0.001
Conduct problems	0.84 (0.79–0.89)***	< 0.001	1.12 (1.04–1.21)**	0.002	1.23 (1.11–1.36)***	< 0.001
Peer relationship problems	0.87 (0.83–0.92)***	< 0.001	1.11 (1.04–1.18)**	0.002	1.08 (0.99–1.18)	0.08

OR = Odds Ratio, CI = Confidence Intervall

*** = p < 0.001; ** = p < 0.01; * = p < 0.05

^a^ SDQ-subscales = Strengths and Difficulties Questionnaire subscales

All associations are adjusted for age, gender and BMI-SDS.

Accurate body size perception was associated with fewer signs of hyperactivity (OR = 0.92, p < 0.001) and fewer peer relationship problems (OR = 0.90, p < 0.001). Hence children who did not perceive their body size correctly, showed more signs of hyperactivity. The strengths of the association did not differ between over – or underestimation one’s body size. Furthermore, accurate body size perception was significantly associated with fewer emotional problems (OR = 0.95, p = 0.03). Here, the reverse association was stronger for children and adolescents who overestimated their body size. We found no significant association between an accurate body size perception and prosocial behaviour or conduct problems (both p > 0.05), as shown in Table 4.

**Table 4:** Association between behavioural and emotional difficulties and body size perception

SDQ-subscales^a^	Accurate self-perception		Over-estimation		Under-estimation	
	OR (95% CI)	p-value	OR (95% CI)	p-value	OR (95% CI)	p-value
Prosocial behaviour	0.99 (0.95–1.05)	0.94	0.89 (0.80–0.99)*	0.03	1.05 (0.98–1.12)	0.15
Hyperactivity	0.92 (0.89–0.96)***	< 0.001	1.08 (0.99–1.18)	0.79	1.08 (1.02–1.14)**	0.004
Emotional problems	0.95 (0.91–0.99)*	0.03	1.13 (1.03–1.24)*	0.01	0.97 (0.92–1.03)	0.32
Conduct problems	0.96 (0.90–1.02)	0.20	1.03 (0.90–1.17)	0.68	1.01 (0.94–1.10)	0.73
Peer relationship problems	0.90 (0.85–0.95)***	< 0.001	1.09 (0.97–1.22)	0.17	1.06 (0.99–1.14)	0.08

OR = Odds Ratio, CI = Confidence Intervall

*** = p < 0.001; ** = p < 0.01; * = p < 0.05

^a^ SDQ-subscales = Strengths and Difficulties Questionnaire subscales

All associations are adjusted for age, gender and BMI-SDS.

Greater prosocial behaviour problems (OR = 0.89, p = 0.03) and emotional problems (OR = 1.13, p = 0.01) were significantly associated with overestimating one’s body size. However, we found no significant association between overestimation and hyperactivity, conduct problems and peer relationship problems (all p > 0.05). On the other hand, underestimation of the own body size was only associated with more hyperactivity problems (OR = 1.08, p < 0.05). No other significant associations were found (all p > 0.05).

All statistical models presented in Tables 3 and 4 were checked for interactions between each independent variable and age or gender. However, interactions did not reach statistical significance.

## Discussion

Whereas previous studies mainly focused on body image satisfaction and its association with only one behavioural or emotional difficulty in children and adolescents, nearly nothing is known about body size perception and its relation to different behavioural or emotional difficulties. This study sought to fill the gap by providing an overview on body image satisfaction and body size perception and the association with several behavioural and emotional difficulties in children and adolescents. Furthermore, the differences between gender, age, and weight status and their inter-relation were assessed. Overall, we found that a significant part of German children and adolescents is dissatisfied with their bodies, which was also significantly associated with behavioural and emotional difficulties. Moreover, body size misperception was associated with behavioural and emotional difficulties as well. These findings are important for both scientific research and practical interventions to enhance the positive body image and psychological and physiological well-being of children and adolescents.

### Prevalence of body image disturbances and associations with sociodemographic factors

In our study, more than 60% of the children and adolescents reported being at least slightly dissatisfied with their body image. This is in line with previous studies from different countries [17, 28].

Compared to boys, girls were more dissatisfied with their body image and were more likely to desire a thinner body. Previous research suggested that the influence of media and advertisements encouraged girls to strive for a thin and lean body shape and boys to become more muscular [22].

Adolescents were more dissatisfied than children, which could be partially explained by the physical and psychological changes during puberty [13]. Furthermore, we found that children and adolescents with underweight or normal weight were more satisfied than children and adolescents with overweight or obesity, which is in line with previous research [51]. A possible explanation is the internalization of a thin and lean body image ideal influenced by media, peers, and family [1].

We want to emphasize that there might be children or adolescents who want to decrease body weight but still are satisfied with his/her body if he/she has a positive body image. However, because, in general, overweight and obesity carry a stigma in Western civilizations and, subsequently, affected boys and girls are often bullied [7, 8, 42], we assume the majority experiences being overweight or obese as negative. More so because the included age range is associated with particularly sensitive feelings regarding one’s own body induced by the rapid changes in body composition and appearance [13, 50] during puberty. Therefore, we think it is justified to interpret the difference between perceived and desired body size as a proxy for body dissatisfaction in our study, which is in line with several other studies [26, 38, 40].

In our study, about 68% of all children and adolescents perceived their body size correctly. However, the prevalence of an accurate body perception has considerable variations. Among Chinese children and adolescents, 55.5% perceived their weight correctly [43], whereas only 42.6% of American middle school students showed an accurate perception [32]. In Iran, 40.9% of children and adolescents had an accurate body weight perception [47]. These variations might be explained by cultural differences. Body size misperception is associated with weight-related concerns and unhealthy eating behaviours [39, 66]. Neumark-Sztainer et al. (2002) found that Asian Americans and Native Americans report similar or more weight-related concerns than white girls [39]. These findings could be explained by different, ethnic-specific beauty ideals [9, 39]. Therefore, it can be suggested that the accurate perception of one’s body weight is different across various cultures.

In contrast to previous research [3, 47], we found that girls were more likely to underestimate their body size than boys. Methodological differences between the studies may also play a role, since they used specific questionnaires to assess over – and underestimation of a child’s weight status, whereas we calculated the discrepancy. Furthermore, studies have shown that parents of normal weight, overweight and obese children and adolescents tend to underestimate their child’s weight [19, 27, 49]. The parental underestimation can cause overweight or obese children to regard their weight status as normal [60]. Therefore, children and adolescents underestimate their own body weight, which can lead to unhealthy behaviour like exercising less or eating more calories [32].

Moreover, there has been a dramatically rise in the overweight prevalence among children and adolescents in Germany [33, 48]. Also body positivity has become more present over the last decade [15]. The increasing prevalence of overweight as well as the rise of body positivity could lead to the thinking that being overweight is normal [58].

Finally, we found that being older was associated with an accurate body size perception. A possible explanation is that as children grow older, their self-concept becomes more differentiated and they may have a better understanding of body size and weight [14, 67].

### Association between body image satisfaction and body size perception

Our findings showed that body image dissatisfaction might be affected by body size perception or vice versa. However, body dissatisfaction also occurred in children and adolescents who perceived their body size correctly. This finding is in line with previous research [51]. Interestingly, children and adolescents who desired to be thinner were more likely to have an accurate body size perception than children and adolescents who desired to be larger. Our results also showed, that the desire to be thinner was significantly associated with greater BMI-SDS. Due to our obesity cohort, it is reasonable to suggest, that obese children and adolescents participating in a study were more aware of their body weight and size.

### Association between behavioural and emotional difficulties and body image disturbances

Participants who desired to be thinner or larger than their perceived body image showed more behavioural and emotional difficulties, which is comparable to previous studies [10, 18, 31]. These findings could be explained by the increased usage of social media over the last years [22]. Edited pictures of peers or role models and advertisement may lead to unrealistic body image ideals and unachievable standards [1, 22]. Moreover, the number of friends and the constant social comparison enhances the internalization of a thin/muscular body ideal [29, 57]. This can lead to increased body image dissatisfaction and, furthermore, to low self-esteem, anxiety and behavioural problems [11, 46]. Furthermore, teasing and weight-related bullying is frequent especially among adolescents [61], which leads not only to body image dissatisfaction but also to disordered eating like restraint or bulimic behaviour [35]. Regarding hyperactivity, different studies found that hyperactivity and/or ADHD predict body image dissatisfaction [5, 46]. Additionally, the prevalence of adiposity has been increasing over the last years for children and adolescents diagnosed with ADHD [5]. Body mass might explain the association between body image dissatisfaction and hyperactivity [5].

Finally, it is not surprising that we found body image satisfaction to be associated with fewer behavioural and emotional problems. Peers and family can also be a positive influence. Studies have observed that children and adolescents who had more social support from friends and family showed less body dissatisfaction [4, 64]. More social support indicates a higher self-esteem and less depressive symptoms or emotional problems [64]. Therefore, good social support might explain the association between body image satisfaction and fewer emotional problems.

Our data showed that children and adolescents, who overestimated their body size, also showed more emotional and prosocial behaviour problems. Overestimation might be an expression of body image dissatisfaction, leading to low self-esteem and feelings of depression [47]. Furthermore, Seo and Lee (2013) found that overestimation in girls increases the risk of suicidal ideation which was mediated by depression [54].

Underestimation of one’s body size was related to more signs of hyperactivity. A large German study showed that children and adolescents with more symptoms in hyperactivity were significantly more physically active [62]. Similarly, children and adolescents who were more physically active were more likely to underestimate their body weight [20]. It may be reasonable to suggest that children and adolescents with signs of hyperactivity were more physically active and, therefore, underestimated their body size or weight.

Moreover, underestimation might be a protective factor against depressive symptoms [59]. However, we did not find a respective association. However, unlike our study sample, Thurston et al. (2017) only included children and adolescents with overweight and obesity. Additionally, they did not apply the SDQ but the Center for Epidemiologic Studies-Depression Scale, which measures depressive symptoms over the past week.

Finally, we found perceiving one’s body size correctly was associated with fewer behavioural and emotional difficulties. This might be explained by higher self-esteem toward one’s own body and, therefore, a lower risk for internalizing the thin/muscular body ideal [11].

All of these findings suggest to pay attention to children and adolescents displaying behavioural problems and showing body image disturbances. Children and adolescents should be guided to understand their own body and the development of it during puberty. Schools, doctors and parents should be interested in the education about social comparison, (social) media and other aspects. The findings indicate the need for screenings to detect behavioural and emotional difficulties early and to prevent body image disturbances as risk factors for several health outcomes like eating disorders.

### Strengths and Limitations

The strengths of the present study are the large sample size and the variety of measured behavioural and emotional difficulties in combination with both components of body image disturbances.

Nonetheless, some limitations have to be noticed. First, despite BMI-based measures are widely used and accepted for the assessment of weight status, there are some limitations because it does not differ between fat and lean body mass, which, however, is much more difficult to gauge and was not available in our study. Furthermore, the high prevalence of children and adolescents with obesity due to our obesity cohort could lead to a bias in our results. Second, the SDQ is a screening questionnaire and not a diagnostic instrument. Furthermore, depending on the child’s age, the data on behavioural and emotional difficulties were assessed via either parental report (for 8–10 years old children) or self-report (children over the age of 10 years). Further, we want to point out that we had relatively low values of internal consistency for two SDQ scales (conduct problems, peer relationship problems). Moreover, children and adolescents were not directly asked about their body image satisfaction or body size. Only the use of silhouette scales and the calculation of the discrepancies was used. Therefore, it must be considered that associations between body image dissatisfaction or body size misperception and behavioural and emotional difficulties as well as variations depending on the informant might differ depending on the instrument used to assess data. The FRS was designed by our researchers and were not validated separately as only the number of figures varies and no significant changes were made. However, since other studies used similar scales, we assume that the data can be relied upon. Another limitation is the cross-sectional study design which does not allow causal interpretation.

## Conclusion

The present study shows the alarming prevalence of body image disturbances in healthy German children and adolescents. Body image dissatisfaction and body size misperception are associated with behavioural and emotional difficulties. Moreover, body image disturbances were observed in every gender, age - and weight group. The findings might indicate that behavioural and emotional difficulties implicate body image disturbances, which should be, therefore, be included in diagnostic routines and/or preventive measures.

### Electronic supplementary material

Below is the link to the electronic supplementary material.

Supplementary Material 1

## Data Availability

The datasets generated and/or analysed during the current study are not publicly available due to ethical restrictions. The LIFE Child study is a study collecting potentially sensitive information. Publishing data sets is not covered by the informed consent provided by the study participants. However, every researcher affiliated with a research institution can request data access. Researchers who interested in accessing and analyzing data collected in the LIFE Child study may contact the data use and access committee (Dr. Matthias Nüchter; info@life.uni-leipzig.de).

## List of abbreviations

BMI-SDS: Body mass index – standard deviation score
CI: Confidence Intervall
FRS: Figure Rating Scale
OR: Odds Ratio
SDQ: Strengths and Difficulties Questionnaire
